# Toward peripheral nerve mechanical characterization using Brillouin imaging spectroscopy

**DOI:** 10.1117/1.NPh.10.3.035007

**Published:** 2023-08-26

**Authors:** Vsevolod Cheburkanov, Junwei Du, David M. Brogan, Mikhail Y. Berezin, Vladislav V. Yakovlev

**Affiliations:** a Texas A&M University, Department of Biomedical Engineering, College Station, Texas, United States; b Washington University School of Medicine, Department of Radiology, St. Louis, Missouri, United States; c Washington University, Institute of Materials Science and Engineering, St. Louis, Missouri, United States; d Washington University School of Medicine, Department of Orthopedic Surgery, St. Louis, Missouri, United States

**Keywords:** brillouin, confocal imaging, nerve, *ex vivo*

## Abstract

**Significance:**

Peripheral nerves are viscoelastic tissues with unique elastic characteristics. Imaging of peripheral nerve elasticity is important in medicine, particularly in the context of nerve injury and repair. Elasticity imaging techniques provide information about the mechanical properties of peripheral nerves, which can be useful in identifying areas of nerve damage or compression, as well as assessing the success of nerve repair procedures.

**Aim:**

We aim to assess the feasibility of Brillouin microspectroscopy for peripheral nerve imaging of elasticity, with the ultimate goal of developing a new diagnostic tool for peripheral nerve injury *in vivo*.

**Approach:**

Viscoelastic properties of the peripheral nerve were evaluated with Brillouin imaging spectroscopy.

**Results:**

An external stress exerted on the fixed nerve resulted in a Brillouin shift. Quantification of the shift enabled correlation of the Brillouin parameters with nerve elastic properties.

**Conclusions:**

Brillouin microscopy provides sufficient sensitivity to assess viscoelastic properties of peripheral nerves.

## 1 Introduction

Nerve elasticity is a biologically important but understudied concept. This property refers to the ability of nerves to stretch and deform in response to mechanical stress while maintaining their function and structure.^1^ Nerve elasticity is particularly important in the peripheral nervous system, where nerves are exposed to a wide range of mechanical stresses and strains as they traverse the body and in the setting of trauma.^2^ Nerves’ mechanical properties are also essential for research and development of neural grafts for peripheral nerve regeneration.^3^^,^^4^ Nerve elasticity is influenced by several factors, including the type and architecture of the nerve fibers (motor versus sensory), the composition of the extracellular matrix (ECM) surrounding the nerve, and the activity of specific molecular signaling pathways within the nerve cells.

Sensory or muscle activation pathways in most living organisms involve action potential propagation along axons toward a given synapse or neuromuscular junction. Individual motor and sensory neurons originate from the spinal cord and are bundled into nerve fibers comprised mostly of axons and Schwann cells. Motor neurons originate from the ventral horn and innervate skeletal muscles that allow volitional control of the extremities. Sensory pathways are governed by neurons originating from the dorsal root ganglia located just outside the spinal cord, adjacent to the intervertebral foramina through which spinal nerves exit. Motor and sensory neurons combine to form the spinal cord nerve roots. Major peripheral nerves in mammals (e.g., sciatic nerve, tibial nerve, and ulnar nerve) can be a mixture of motor and sensory neurons or consist of pure motor or sensory components (e.g., sural nerve has mostly sensory neurons). The structural stability and flexibility of the nerves are largely maintained by connective tissues, such as endoneurium surrounding hundred and thousands of individual axons, perineurium that bundles axons together to form fascicles, and epineurium that wraps around the whole nerve. These connective tissues, composed of cells and ECM made of glycoproteins, proteoglycans, and other macromolecules, provide physical support to the nerve and maintain its structural integrity. The connective tissue also helps to regulate axon growth and regeneration and provides a supportive environment for the passage of electrical signals along the nerve. Rich vascular networks in the connective tissue provide neurons with oxygen, glucose, and other molecules to ensure that the neurons have sufficient nutrients to conduct motor or sensory functions.

The complex nerve architecture facilitates physical protection and mechanical rigidity as well as blood supply to encapsulated neurons and connective tissue. Nerve trauma can produce a spectrum of injuries ranging from a conduction block to complete transection.^5^ Nerve injury due to nerve stretching is also common, although nerves possess some degree of elasticity. Nevertheless, when their limit is exceeded, the nerve can be torn apart, resulting in mild to severe nerve injuries. Stretch and traction are common causes of brachial plexus, radial, and peroneal nerve injuries associated with injuries such as fractures. Factors that affect the healing process include the location of the nerve, metabolic conditions, aging, chemotherapy drugs, and others. Traditional methods for measuring the damage of the nerve are largely based on histomorphometry to visualize the geometry of the axons and quantify clinically important parameters, such as the degree of myelination and presence of inflammation. Still, these techniques are limited in clinical applicability as they do not give any information in real time to surgeons assessing injured nerves. Having a method that can measure the elastic and other mechanical properties of healthy and stretched nerves *ex vitro* and even *in vivo* will add additional information essential for understanding degree of peripheral nerve injury and aid in clinical decision making. Being able to determine mechanical properties of nerve damage with a noninvasive tool in different conditions is crucial as previous studies^6^^,^^7^ suggest that different mechanical properties exist in nerves under different evaluation conditions.

Brillouin imaging spectroscopy is an emerging imaging technique to measure mechanical properties of biological tissues such as superficial cancers,^8^ neurons,^9^^,^^10^ eye,^11^^–^^15^ brain,^16^ vasculature,^17^ and bones^18^ with high spatial resolution. This technique is based on the Brillouin scattering effect, which occurs when light interacts with acoustic waves in a material. In Brillouin imaging spectroscopy, a laser beam is directed at a sample, and the resulting Brillouin scattered light is collected and analyzed. The frequency of the scattered light is dependent on the mechanical properties of the material, such as its elasticity and density. By analyzing the frequency shift of the scattered light, it is possible to determine the mechanical properties of the sample at a high spatial resolution. One advantage of Brillouin imaging spectroscopy is that it is non-invasive, so it does not require the use of contrast agents or other invasive procedures. This makes it a valuable tool for studying the mechanical properties of biological tissues *in vivo* in clinical models and potentially evaluating the damage in clinical settings.

Peripheral nerve damage is detrimental to affected patients, and it is crucially important to imaging, understanding, and evaluating the scale of damage to design a proper treatment strategy. To achieve these goals, one requires a tool for non-invasive evaluation of local mechanical properties of the peripheral nerve. Brillouin spectroscopy, as a non-invasive, high-resolution technique, is a suitable candidate. To our knowledge, Brillouin spectroscopic imaging of peripheral nerves has not been previously reported. Due to this, our group focuses on applying Brillouin microspectroscopy to *ex vivo* fixed peripheral nerve elasticity evaluation. Results reported in this paper are the first recorded step in developing a new method for clinical evaluation of nerves through characterization of Brillouin parameters in a murine sciatic nerve model.

## 2 Materials and Methods

### 2.1 Experiment Design

**Fig. 1:**
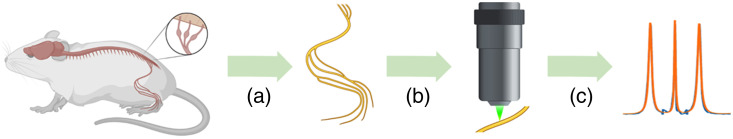
Workflow of Brillouin microscopy imaging for assessing peripheral nerves. (a) Sciatic nerve is harvested from a sacrificed mouse. (b) Nerve sample is chemically fixed and prepared for imaging. (c) Nerve sample is imaged with Brillouin microspectroscopy. Created with Ref. 19.

In this study, we evaluate the feasibility of Brillouin spectroscopy to assess the mechanical properties of peripheral nerve *ex vivo* with the ultimate goal of using this emerging technology to assess viscoelastic properties of peripheral nerves *in vivo*. The workflow of the experiment is shown in Fig. 1.

To determine the effect of nerve injury on the mechanical properties assessed by Brillouin imaging, we designed an experiment to assess both healthy and injured nerves. For the healthy nerve sample, the harvested sciatic nerves were suspended in free space without any applied stress. In the injured nerve model, the harvested sciatic nerve was compressed in a fixture mount, as described below, causing visible mechanical deformation of the tissue.

**Fig. 2:**
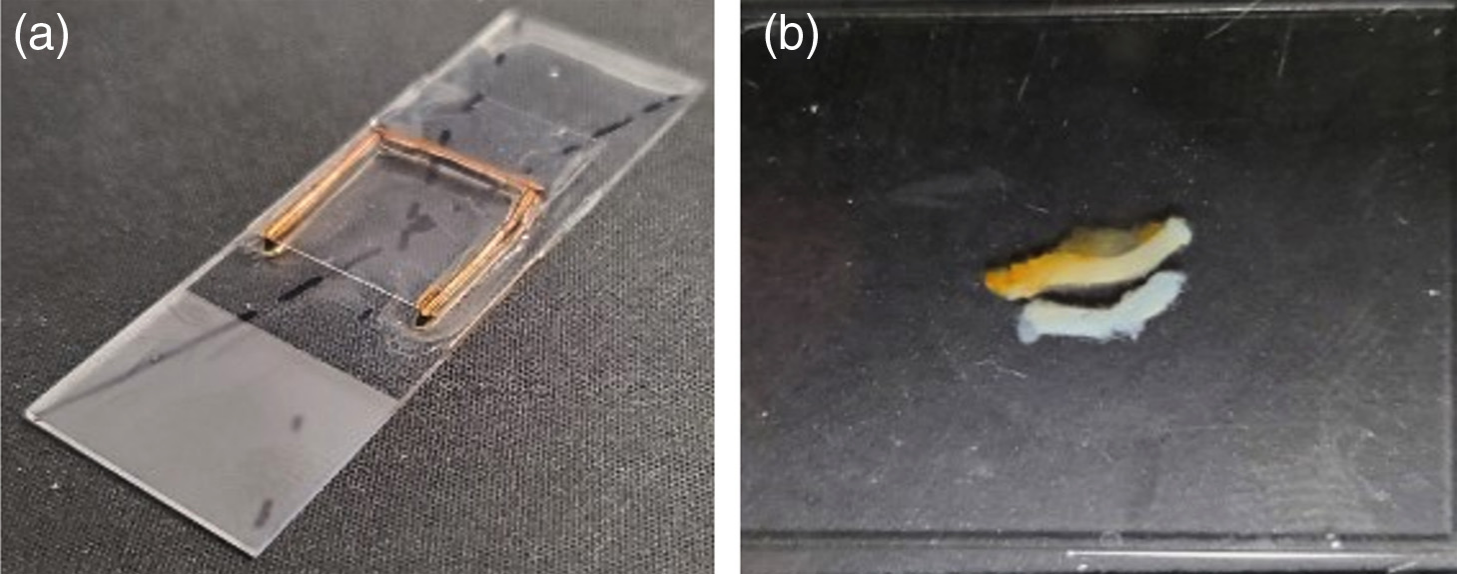
(a) A photographic image of a custom-built open sample holder. (b) A photographic image of a lab-grade closed sample holder.

Orthogonal techniques were utilized to evaluate the responses from both intact and damaged nerves for Brillouin imaging, each designed to prevent sample deterioration and rapid evaporation of submersion media. For the healthy nerve, a partially open mount was designed [Fig. 2(a)], and for the injured nerve, a completely enclosed fixture was utilized [Fig 2(b)].

The open holder was designed and assembled in such a way that the sample, when placed inside, was free of any external forces that could alter its density or other properties. To avoid dehydration of the sample, the volume inside the holder was filled with phosphate-buffered saline (PBS) solution.

The closed fixture consisted of a quartz concave slide and a quartz microscope coverslip. This sample holder, albeit sealed from the atmosphere and filled with PBS, demonstrated visible compression of the nerve samples.

Validation of the interrogated region location was achieved through analysis of the signal from the Raman channel of the spectrometer, described in the text prior. The imaging plane field was scanned by means of sample table movement with fixed increments exceeding the spatial resolution of the spectrometer.

### 2.2 Sample Preparation

All animal studies were conducted in compliance with the Washington University Institutional Animal Studies Committee and NIH guidelines. C57BL/6 male mice (Charles River) were used in these experiments. Mice were housed in a central animal care facility and provided with food and water ad libitum. Animals were kept in standard cages at room temperature and a 12:12 day/night light cycle. Each mouse was anesthetized with 2% (v/v) inhalational isoflurane and then sacrificed by cervical dislocation. The sciatic nerves on both sides were harvested through a mid-thigh incision.^20^ A small vertical incision was made along the thigh, skin was retracted laterally, and the muscles of the posterior thigh were split with scissors to expose the sciatic nerve. The nerve was then gently lifted with forceps and removed by carefully cutting the connecting tissues to avoid deformation.

A murine model is commonly used to study neuropathic pain^21^^,^^22^ and nerve regeneration.^23^ This model is well studied and yields highly replicable results. The short sciatic nerve length is detrimental in some mechanical evaluation studies, whereas microscopic evaluation of small sections of sciatic nerve is not detrimental, but rather advantageous. In addition to this, the murine model is very common in chemotherapy response evaluation,^24^ showing that such treatment can induce detrimental systemic response and peripheral neuropathy.^25^

One of the nerve samples was subjected to mechanical physical damage *ex vitro*, which decreased the circumference of the sample by ∼20% through the removal of the surrounding connective tissue. This sample is referred to as the “damaged” nerve sample.

Between measurements, nerve samples were stored in the fridge at 4°C in separate vials submersed in the PBS solution.

### 2.3 Brillouin Imaging Spectrometer

**Fig. 3:**
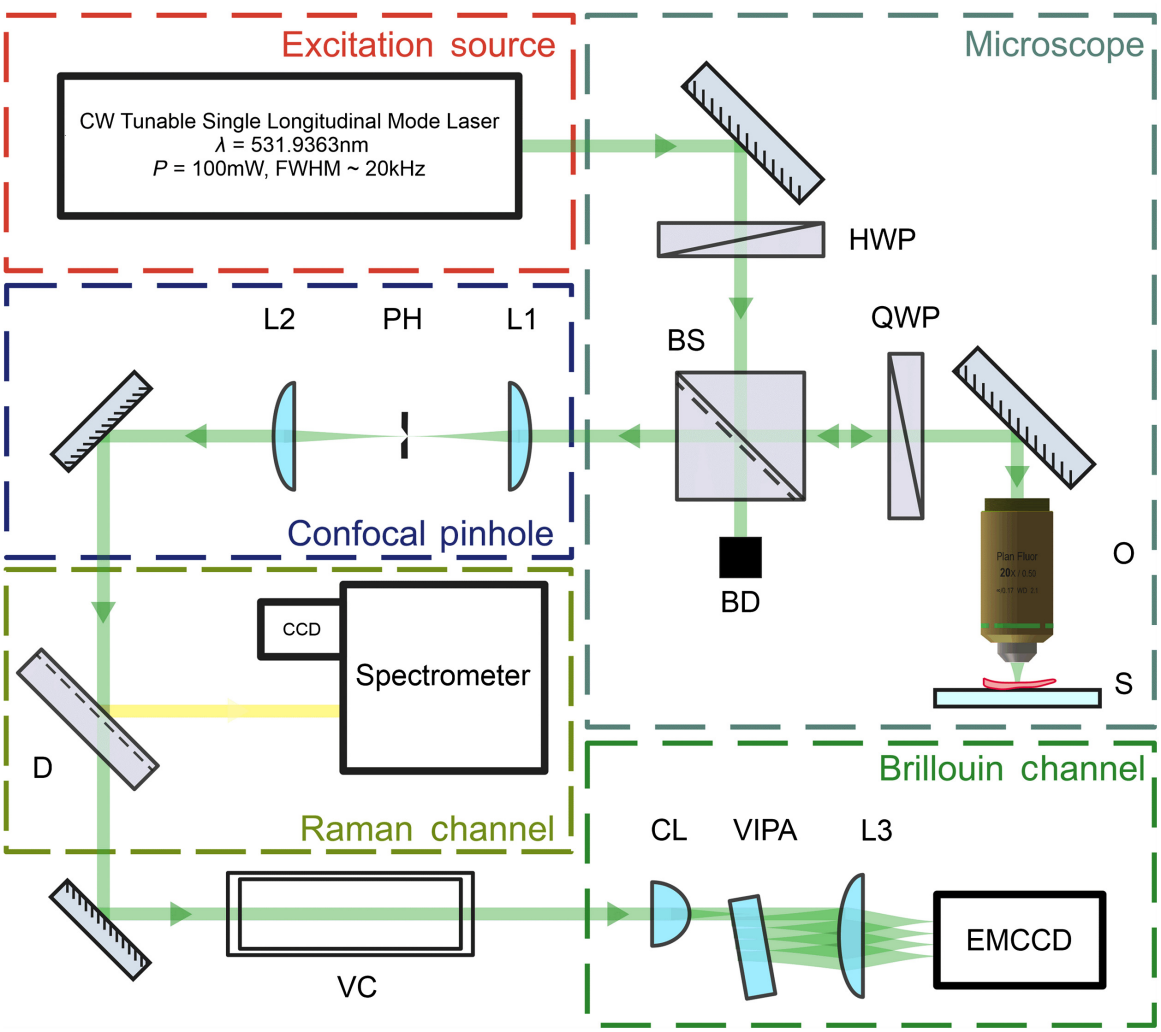
Combined confocal Brillouin–Raman microspectrometer. HWP, half-wave plate; QWP, quarter-wave plate; BS, polarizing beamsplitter cube; BD, beam dump; O, microscope objective lens; S, sample; L, plano-convex spherical optics; PH, precision pinhole; D, dichroic mirror; VC, iodine vapor cell; CL, cylindrical lens; VIPA, virtually imaged phase array etalon.

This study was performed using a custom-built combined confocal Brillouin–Raman spectrometer (Fig. 3). The detailed description of the setup is provided elsewhere.^26^^–^^28^ The confocal microspectrometer was optimized to acquire the Brillouin signal from highly scattering samples, which potentially makes it possible to image exposed peripheral nerves in the depth of the tissue (10 to 70  μm).

Inelastic scattering was induced using a tunable single-mode 532 nm laser [1064 nm 20 kHz linewidth tunable single mode laser diode (NKT Photonics Koheras Adjustik), seeding an Ytterbium doped fiber amplifier (NKT Photonics Koheras Boostik HPA), and generating 100-mW of 532 nm in a MgO:PPLN crystal (Covesion Ltd.)] shown in a red dashed line box in Fig. 3. Both the excitation and back-scattered signal collection were achieved using CFI60 Nikon microscope objective lenses with a varying numerical aperture (NA) and magnification values. The laser power delivered to the sample was finely controlled by a combination of HWP and BS and did not exceed the 6-mW limit before the objective lens, which has about 70% transmission at the wavelength of 532 nm. The collected and collimated signal was diverted using polarization plane rotation via a double pass through a QWP to the spatial filter composed of a confocal pinhole assembly (blue dashed line box in Fig. 3). The pinhole size was selected to be less than a single Airy unit.

Residual elastically scattered photons were removed from spectra using intrinsic molecular absorption in a heated iodine cell and was then imaged into a custom-built high-resolution spectrometer, which is schematically shown in a green dashed line box (Brillouin Channel) in Fig. 3. A virtually imaged phase array (VIPA) etalon (FSR 29.98 GHz @ 532 nm, OP-6721-3371-2, Light Machinery, Inc.) was used as a dispersive element in this spectrometer. Brillouin spectra were acquired using a water cooled electron-multiplying charge coupled device (EMCCD) (Andor Newport 970, Oxford Instruments), and a longer wavelength signal was steered into a commercial Czerny–Turner spectrometer (Andor Shamrock 303i, Oxford Instruments) fitted with a charge coupled device (CCD) camera (Andor Newport 940P, Oxford Instruments).

The Brillouin spectrometer was calibrated with 10,000 data points. Brillouin standard spectra were acquired from neat acetone sample stored in a sealed quartz cuvette at 37°C heated up with a microscope slide warmer. The mean value for the Brillouin shift was 5.712 GHz with a standard deviation of 0.018 GHz.

The Raman channel was utilized to determine the region of the sample that we are evaluating. The system can acquire optical spectra in the range of 530 to 660 nm. Either of the three metrics are used to determine the sample location: residue backscattering signal, fluorescence signal, or Raman signal. The data acquired from the so-called Raman channel form surface plots, with the height of the surface encoding the spectral peak’s position. We validate nerve location in the field of view of the microscope using the residual backscattering signal.

## 3 Results and Discussions

### 3.1 Non-Compressed Nerve Tissue Sample

**Fig. 4:**
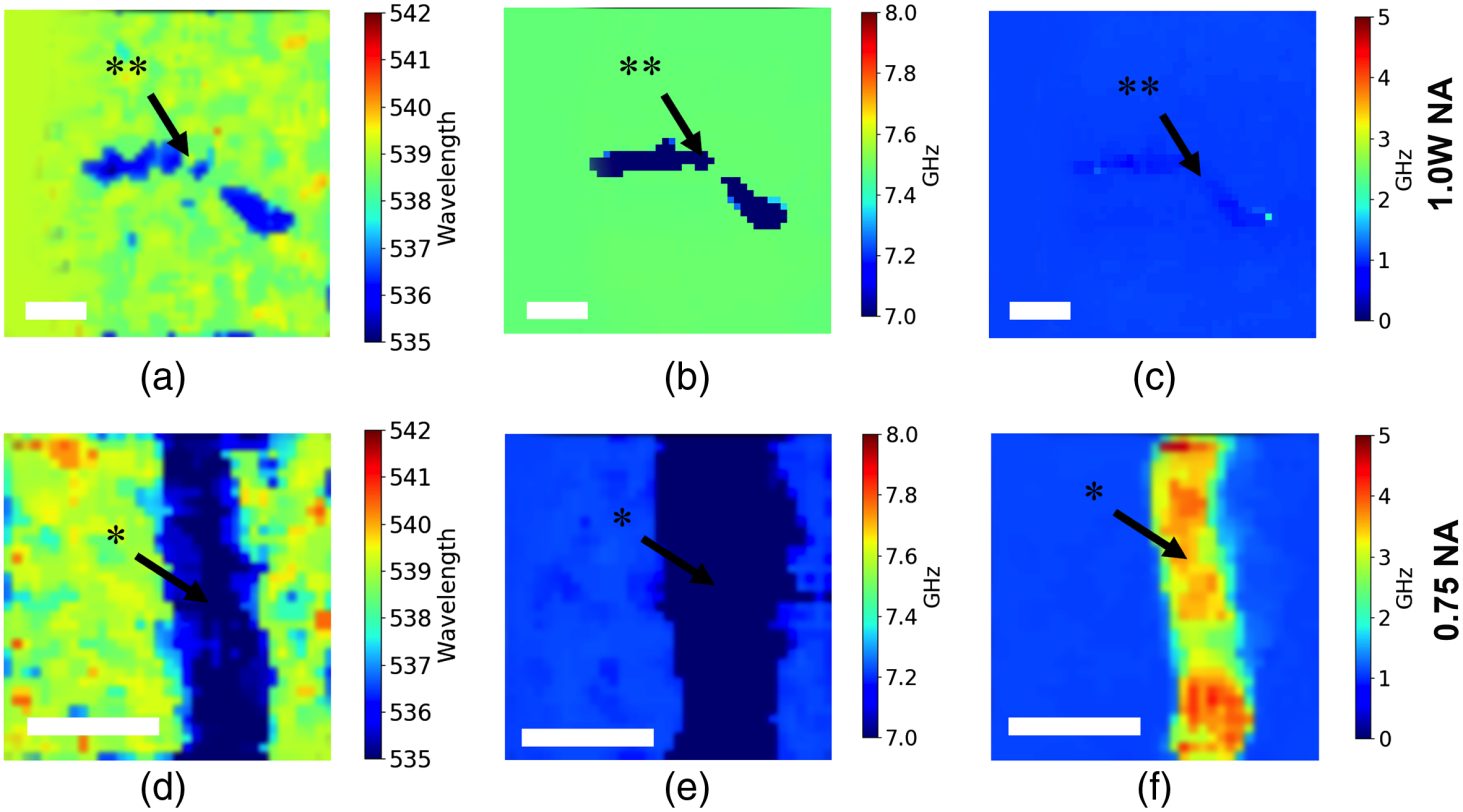
Non-compressed nerves imaged with NA=1.0 objective (top row), pixel size is 220  μm, image size is 11  mm×11  mm; and NA=0.75 objective (bottom row), pixel size is 150  μm, image size is 5.25  mm×5.25  mm. (a), (d) Signal from the Raman channel; (b), (e) Brillouin shift (elasticity) image. (c), (f) Brillouin line FWHM (viscosity) image. White bar represents 2 mm. * represents intact sample; ** represents damaged sample.

Two nerve samples were scanned in the open sample holder to minimize the external disturbance. Data acquired with both NA=1.0 (water immersion) and NA=0.75 (air) objectives are shown in Fig. 4 and show that the Brillouin shift within the sample’s area is lower that than the one from the surrounding media. These data suggest that the nerve region is softer than the surrounding PBS solution. At the same time, the Brillouin linewidth [full width at half maximum (FWHM)] for nerves displayed lower values than the surrounding environment for the damaged sample and higher values for the intact sample, suggesting a lower viscosity of the interrogated region with respect to the immersion media.

Damaged nerve data are presented in Figs. 4(a)–4(c), and the intact nerve is shown in Figs. 4(d)–4(f). According to the Brillouin shift values, both samples were softer than the surrounding environment with the average shift values being 6.48±0.35  GHz for damaged and 6.68±0.44  GHz for intact nerve samples. Brillouin line FWHM values, however, suggest that the viscosity values of the two samples are significantly different. FWHM value from the intact nerve is 0.983±0.0816  GHz and from the damaged nerve is 3.14±0.34  GHz. This can be potentially explained by the lack of external tissues surrounding the neuronal fibers.

We should also note that the Brillouin shift value from the surrounding media is somewhat different for the two measurements. The immersion media, shown in Fig. 4(b), showed 7.48±0.01  GHz, and in Fig. 4(e), the shift value is 7.22±0.02  GHz. This is due to a slight variation of the water temperature.^26^

As shown in Fig. 4, the non-compressed nerve displays a lower Brillouin shift value than the external environment (PBS), which can be explained by the nerve’s low density.

### 3.2 Compressed Nerve Tissue Sample

**Fig. 5:**
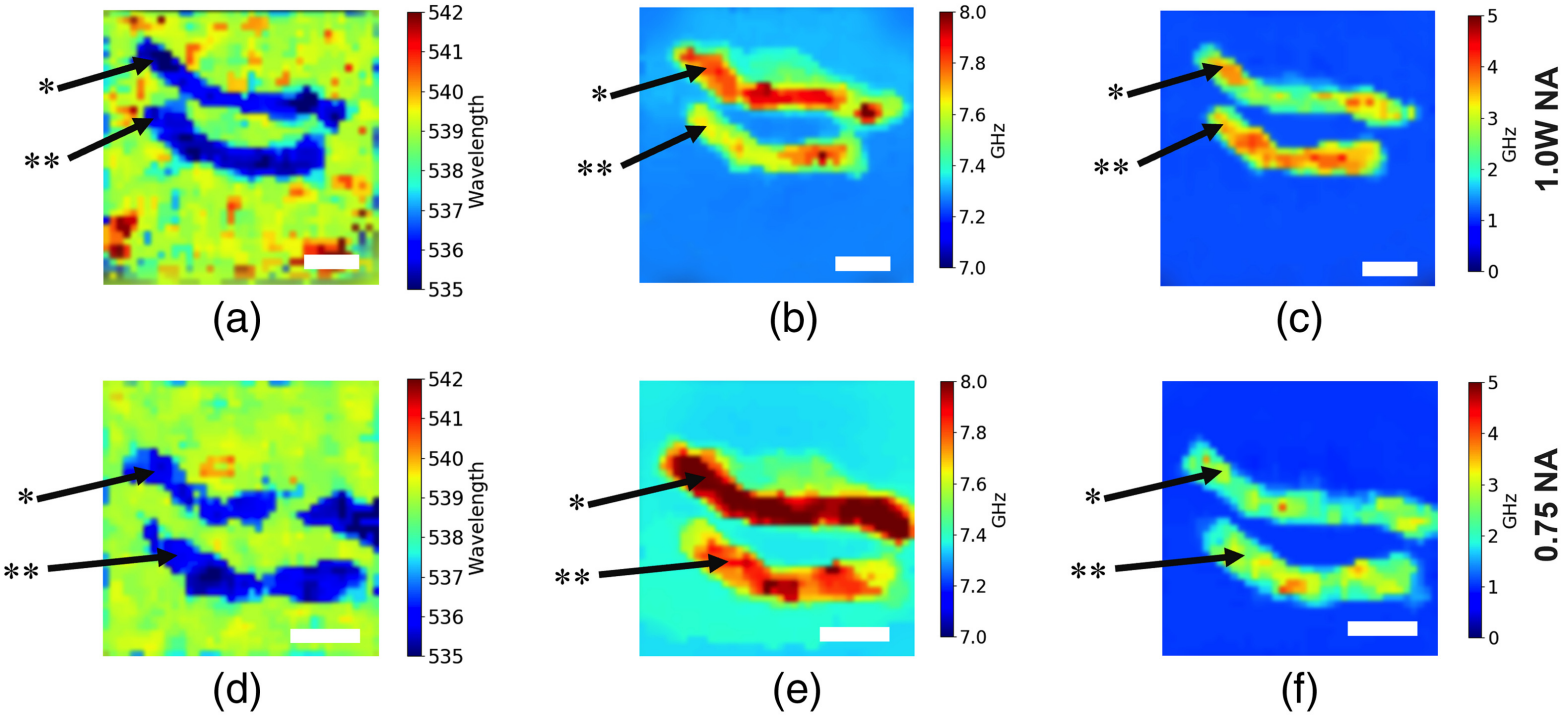
Compressed nerves imaged with NA=1.0 objective (top row), pixel size is 250  μm, image size is 10  mm×10  mm; and NA=0.75 objective (bottom row) pixel size is 200  μm, image size is 8  mm×8  mm. (a), (d) Signal from Raman channel, (b), (e) Brillouin shift (elasticity) image, (c), (f) Brillouin line FWHM (viscosity) image. * represents intact sample, ** represents damaged sample. White bar represents 2 mm.

To further elucidate the mechanical properties of nerves, the nerve tissues were imaged in the closed holder with a compressive force applied to simulate the external stress applied to a nerve. The intact nerve on the top (Fig. 5) had no myelin removed, whereas the one on the bottom underwent mechanical damage, as described in the previous section.

The images were acquired with NA=1.0 and NA=0.75 objectives and are presented in Fig. 5. In contrast with previous (non-stressed) measurements, the nerve structure displayed Brillouin shift values significantly higher than surrounding media, suggesting that stressed nerves are stiffer than immersed media. On the other hand, the Brillouin line FWHM images suggest that nerves are now more viscous than the PBS solution.

A statistical analysis of imaging data presented in Figs. 5(a)–5(c) shows that the intact nerve displays a higher Brillouin scattering shift value (7.78±0.08  GHz with 1.0 NA and 7.95±0.05  GHz with 0.75 NA) than the immersion media and damaged nerve (7.67±0.05  GHz with 1.0 NA and 7.78±0.05  GHz with 0.75 NA). Brillouin line FWHM values from the damaged sample are higher (3.58±0.19  GHz with 1.0 NA and 2.85±0.31  GHz with 0.75 NA) than the intact sample (2.89±0.44  GHz with 1.0 NA and 2.38±0.35  GHz with 0.75 NA). This can be explained, in part, by connective tissue increasing under external load, causing an increase in stiffness and a decrease in viscosity. One possible explanation of the observed changes of the Brillouin spectra from mechanically stressed tissues is related to a change in the sample’s density. It is also plausible that mechanical compression alters optical properties of the samples being imaged. Further investigation is required to interpret this observation.

Similar to the experimental results with the non-compressed sample, we observed a slight variation in the Brillouin shift from the media caused by the sample warming from 4°C to 20°C. We believe that this error is insignificant because the relation between the sample shift and FWHM values remains the same regardless of external temperature variations.

It is important to point out that tissue fixation is known to alter viscoelastic properties of biological samples^29^ and neuronal tissues in particular.^30^ However, comparing samples under the same environmental and imaging conditions yields insightful information about tissue’s viscoelastic properties.

## 4 Conclusions

Peripheral nerves are located throughout the body and can be subject to significant mechanical stress and strain due to their size and location. For example, nerves located in the limbs can be subjected to tension or compression during movement or pressure from surrounding structures. Due to nerve elasticity, these physiological amounts of stress and strain are typically well-tolerated by peripheral nerves and can even be beneficial for nerve health as they can help to promote nerve growth and regeneration. However, excessive or prolonged stress and strain can lead to nerve damage and loss of elasticity, which can result in pain, numbness, weakness, or other symptoms. In this study, we applied Brillouin microspectroscopy for a characterization of peripheral nerve viscoelastic properties.

Our proof-of-principle study demonstrates the promising potential of this technique.^31^ We found that compression of the nerve tissue leads to a significant change in viscoelastic parameters, suggesting stiffening of the nerve sample. These results clearly demonstrate unique features and capabilities of Brillouin spectroscopy, opening an opportunity for preclinical and clinical evaluations of nerve elasticity.
